# Integration of Augmented Reality and Brain-Computer Interface Technologies for Health Care Applications: Exploratory and Prototyping Study

**DOI:** 10.2196/18222

**Published:** 2022-04-21

**Authors:** Anya Andrews

**Affiliations:** 1 Department of Internal Medicine College of Medicine University of Central Florida Orlando, FL United States

**Keywords:** digital health, augmented reality, brain-computer interface, health professional education, clinical performance support, interprofessional teamwork, patient education, mHealth, mobile health, technology integration

## Abstract

**Background:**

Augmented reality (AR) and brain-computer interface (BCI) are promising technologies that have a tremendous potential to revolutionize health care. While there has been a growing interest in these technologies for medical applications in the recent years, the combined use of AR and BCI remains a fairly unexplored area that offers significant opportunities for improving health care professional education and clinical practice. This paper describes a recent study to explore the integration of AR and BCI technologies for health care applications.

**Objective:**

The described effort aims to advance an understanding of how AR and BCI technologies can effectively work together to transform modern health care practice by providing new mechanisms to improve patient and provider learning, communication, and shared decision-making.

**Methods:**

The study methods included an environmental scan of AR and BCI technologies currently used in health care, a use case analysis for a combined AR-BCI capability, and development of an integrated AR-BCI prototype solution for health care applications.

**Results:**

The study resulted in a novel interface technology solution that enables interoperability between consumer-grade wearable AR and BCI devices and provides the users with an ability to control digital objects in augmented reality using neural commands. The article discusses this novel solution within the context of practical digital health use cases developed during the course of the study where the combined AR and BCI technologies are anticipated to produce the most impact.

**Conclusions:**

As one of the pioneering efforts in the area of AR and BCI integration, the study presents a practical implementation pathway for AR-BCI integration and provides directions for future research and innovation in this area.

## Introduction

Augmented reality (AR) and brain-computer interface (BCI) technologies are among the most promising technologies to date offering to revolutionize human-computer interaction in health care and health professional education. AR provides a mixed user experience where virtual and real elements seamlessly coexist to allow the user to see the real world supplemented by virtual objects and data. Most modern AR implementations represent a fusion of computer-generated imagery and real environment using a head-mounted display (HMD) or goggles typically used in gaming, maintenance training, rehabilitation, and surgical performance support. An HMD allows the users to maintain a clear line-of-sight alignment with real elements in the actual environment, which can be beneficial in any data-intensive setting [1]. This is particularly important in clinical environments where a physician’s situational awareness depends on multiple sources of information and simultaneously maintaining effective communications and eye contact with members of the clinical team as well as the patients [1,2].

The BCI represents a communication pathway between the brain and a computer device using a variety of biosensors that gather and interpret the signals from body and mind to enable neural controls over computer functions. The BCI has been used in a wide variety of applications, including rehabilitation, robotics, entertainment, and virtual reality [3-6]. No longer seen as a purely assistive technology, BCI has been gaining interest as a noninvasive physiological observation mechanism applicable to health care and education settings [7]. AR provides an opportunity to integrate feedback into a real-world environment and enhance a user experience by advancing human-computer interaction capabilities, while the BCI enables a new hands-free interaction modality and provides information about the user’s mental state, which supports adaptive training and performance improvement [8,9].

While there has been a growing interest in the AR and BCI technologies in the recent years, the combined use of these technologies remains a relatively uncharted territory both in research and practice. The last decade has brought significant advancements in the area of AR and BCI technologies; however, both of them still exist in a relative isolation from each other. While the idea of bringing these two technologies together has prevailed among futurists, technology enthusiasts, and government research silos for quite some time, it still remains a fairly unexplored area, which had been associated with the realm of science fiction requiring paradigm shifts in digital innovation dynamics [10].

As the researchers and consumers are starting to recognize the benefits for combining BCI and AR fields, this interest continues to fuel the innovation around improved technology interaction and visualization capabilities. There is a growing body of literature suggesting the potential to revolutionize health care through the use of emerging AR and BCI technologies, with a few intriguing examples starting to demonstrate the applicability of these emerging technologies in a variety of health care contexts; for example, surgery, ophthalmology, elderly care, sensory system rehabilitation, and others [8,11-13]. Despite the growing interest in the AR and BCI technologies, the research in this area is currently somewhat fragmented, and the awareness of the true potential of AR and BCI is still rather limited [1,14].

A combination of AR and BCI technologies can offer an enhanced user experience both for patients and health care professionals, particularly from procedure-intensive specialties, by allowing them to interact with a mix of real and virtual objects, contextual elements, and each other, while using the BCI as an additional communication vehicle, besides the spoken word and hand gesture traditionally used in AR. For instance, through interactive 3D visualizations, an AR component can be used to help a health professional explain a disease or a medical procedure to a patient during a clinical encounter or provide visual cues to a physician during a complex procedure, while a BCI component can simultaneously use biosensors, such as an electroencephalogram (EEG), to enhance the range of options for performing clinical tasks through a combination of verbal, tactile, and neural triggers as well as provide new information about the user’s mental state.

This paper presents the results of a recent effort aimed to advance an understanding of how AR and BCI technologies can work together to transform modern health professional education and clinical practice by providing practical mechanisms to support the established principles of patient-centered care [15] as they relate to patient safety, effective patient-provider communication, shared decision-making, and patient education. The aim of this study was to explore the integration of commercially available wearable AR and BCI technologies that can be applied in medical education, clinical practice, and other areas to address a variety of real-world challenges in health care. The study produced a novel integrated AR-BCI technology solution, which was demonstrated within the context of practical use cases focused on health professional education and clinical performance support.

## Methods

### Methods Overview

The study methodology included an environmental scan and analysis of modern AR and BCI technologies, a use case analysis of practical applications for combined use of AR-BCI technologies in health care, and the development of a proof-of-concept AR-BCI technology integration prototype situated within the modeled use case scenarios, as summarized in the following paragraphs.

### Environmental Scan and Analysis of Modern AR and BCI Technologies in Health Care

The environmental scan [16] and analysis component constituted a broad-scale review of existing applications of AR and BCI technologies in health care through literature review, research and industry reports, technology demonstrations in health care settings, and other sources. The literature review included publications identified from health care and technology research databases (eg, PubMed, IEEE, EBSCO, and others) using the following Medical Subject Headings (MeSH) terms: “augmented reality,” “brain-computer interfacing,” “healthcare,” “clinical performance support,” and “health professional education.” The survey of industry reports and technology demonstrations was performed at a number of major technology innovation venues (eg, Consumer Electronics Show, Interservice/Industry, Simulation, Training, and Education Conference, Healthcare Information and Management Society, International Meeting for Simulation in Healthcare, and others). The environmental scan revealed the strong potential for bringing the AR and BCI technologies for health care applications, particularly within the context of complex medical interventions and treatment planning (eg, surgery, invasive testing procedures, intensive therapies, and others). Leveraging the combination of AR and BCI in such cases would help improve communication and shared decision-making between providers and patients as well as members of an interprofessional team. At the same time, the environmental scan has confirmed that while the use of AR and BCI technologies in health care is growing, their combined use remains an unexplored area where the majority of innovations currently reside in research laboratories and apply to a limited range of clinical applications and disease conditions.

### Use Case Analysis of Practical Applications for Combined AR-BCI Technologies in Health Care

To explore the potential for the combined use of AR and BCI technologies in health care, a use case analysis technique [17] was used, which helped identify the requirements for the AR-BCI within the context of practical health care applications. Through multidisciplinary collaboration with experts from health professional education, clinical sciences, and computer and cognitive sciences, a series of use cases were developed, which focused on the following key areas where the combined use of AR-BCI technologies can produce the most impact:

- Medical or health professional education
- Patient education
- Patient-provider communication
- Shared decision-making
- Clinical performance support
- Interprofessional teamwork

These use cases provided the basis for modeling the simulation scenarios used to demonstrate and validate the AR-BCI proof-of-concept technology, which is described in the *Results* section.

### AR-BCI Technology Integration Prototype Development

The prototype development effort involved a proof-of-concept integration of AR-BCI technologies with the intent of demonstrating the potential of the combined technologies within the context of the practical use cases and serve as a test bed for future use case scenarios and implementation. The prototype development effort focused on the integration of commercially available consumer AR and BCI devices to minimize the common barriers associated with the use of specialized technologies, which frequently stand in the way of technology implementation and adoption.

The proof-of-concept AR-BCI integration was performed using Microsoft HoloLens as the AR technology component and NeuroSky Mind Wave 2 as the BCI component. While neither of the two devices were designed to work together “out of the box” in a plug-and-play fashion, they do come equipped with a software development kit and application programming interface components, which make integration with other technology platforms and devices possible in principle. It is important to note, however, that coupling these devices involved a technology development and programming effort to create a software interface to enable the communication between them. A WebSocket relay server was implemented as an intermediary component between HoloLens and NeuroSky Mobile Wave 2 because both devices support the internet connection over an HTTPS internet protocol. The WebSocket protocol was selected on the basis of its effective real-time performance as a relay messenger, which, in this case, listened for the messages from the BCI device (NeuroSky) and relayed them to the connected AR device (HoloLens). As part of the prototype validation efforts, this technology integration method was also successfully coupled with other consumer-grade BCI or neurosensing devices, such as MUSE, and is currently being extended to other AR devices, such as Magic Leap and MERGE.

## Results

The principal outcome of this exploratory assessment and prototype development is that the new technology interface that resulted from it enables the coupling and communication between these devices—a capability that previously did not exist. Specifically, the study resulted in a proof-of-concept integration of mainstream consumer AR and BCI technologies and development of a novel integrated technology platform to demonstrate their potential within the context of medical education and clinical performance support use cases and serve as a test bed, based on which future developments can be performed and evaluated.

**Figure 1:**
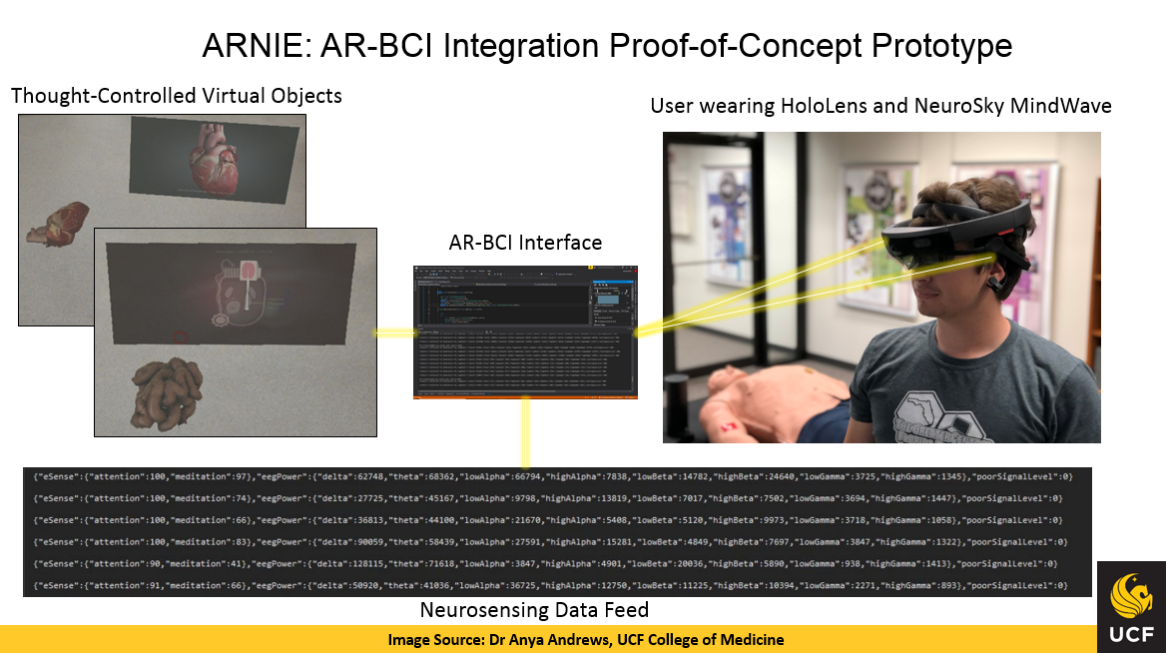
Augmented reality and brain-computer interface (AR-BCI) proof-of-concept integration prototype: medical education use case. ARNIE: Augmented Reality and Neurosensing Interaction Environment, UCF: University of Central Florida.

Named “*Augmented Reality and Neurosensing Interaction Environment (ARNIE)*,” the resultant solution represents a flexible AR-BCI interface platform and a technology test bed that is specifically intended for consumer-grade devices and is technology brand–agnostic. This solution is designed to enable an enhanced learning experience for health professionals in training and enhanced clinical experience for patients and physicians. The ARNIE platform currently enables thought-controlled manipulation of multiple virtual and data objects in AR similar to a simple computer “mouse click.” Figure 1 illustrates a working model of the ARNIE system within the context of a medical education use case represented via two learning modules—one focused on the cardiovascular disease and the other one on Crohn disease.

The ARNIE system enables the 3D models visualizing a human heart and a gastrointestinal tract to be controlled by a neurosensing BCI component using a unique communication protocol developed to enable coupling of AR-BCI components. As pictured in Figure 1, the student demonstrates wearing an AR headset integrated with a BCI headband and controls the virtual objects—that is, the human heart and gastrointestinal tract—in the AR environment entirely with his thoughts. Specifically, by concentrating on a particular virtual object—for example, “the heart” presented in the AR environment—the student’s EEG waves are captured by the BCI headband, and upon reaching a predetermined attention measure threshold, send a signal using the communication protocol to launch a training video illustrating the functions of the cardiovascular system.

**Figure 2:**
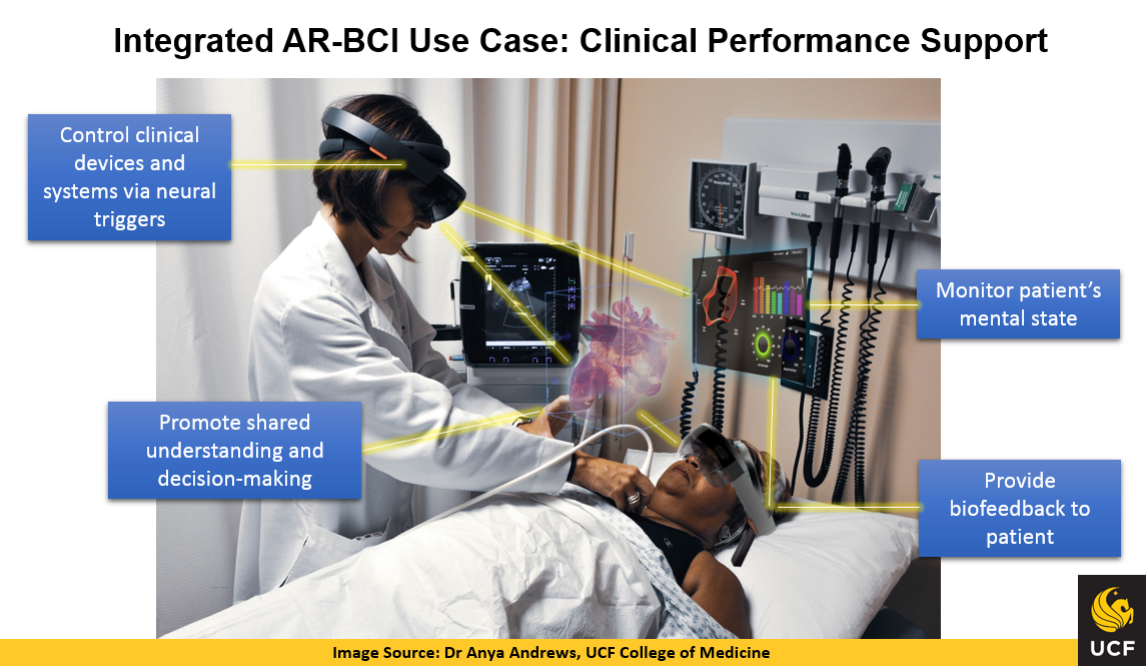
Integrated augmented reality and brain-computer interface (AR-BCI) use case: clinical performance support. UCF: University of Central Florida.

Besides the education and training use cases, the potential of the integrated AR-BCI solution was explored within the context of clinical performance support modeled in a health care environment. Thus, Figure 2 below illustrates a clinical scenario where the integrated AR-BCI capability would allow the physician performing a clinical procedure (eg, an ultrasound of the heart) to control certain clinical devices and systems using neural triggers (ie, attention and concentration), use shared visualizations with the patient (also wearing the AR-BCI devices) to promote effective communication and shared decision-making, while also maintaining enhanced clinical awareness of the patient’s mental state enabled by the patient’s BCI component, which can also be used to provide biofeedback to patient, as needed.

**Figure 3:**
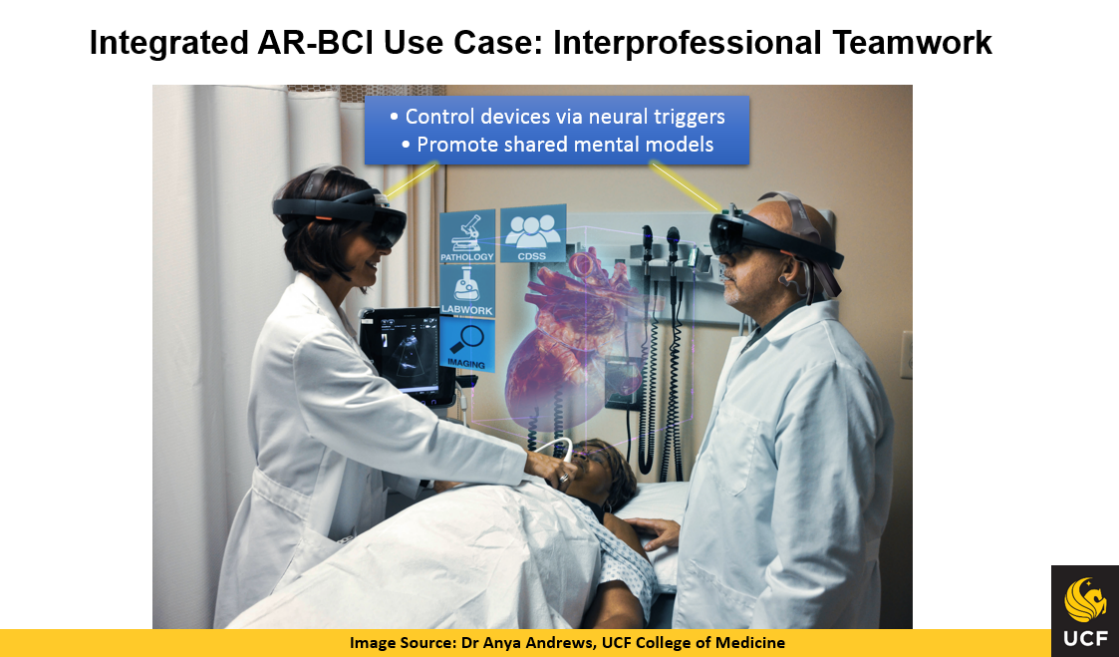
Integrated augmented reality and brain-computer interface (AR-BCI) use case: interprofessional teamwork. UCF: University of Central Florida.

The clinical applications of the integrated solution can also augment inteprofessional teamwork by allowing members of the health care team to control devices and data in the clinical environment via neural triggers, which would help maintain situational awareness and promote shared mental models and shared decision-making as illustrated in Figure 3.

Shared mental models are shared cognitive structures that enable members of an interprofessional team to function collaboratively through implicit coordination while performing clinical tasks that require coordination, cooperation, and mutual support. Shared mental models represent a key prerequisite for shared decision-making in inteprofessional teamwork.

The medical education, clinical performance support, and interprofessional teamwork use case scenarios described above were modeled within the academic technology research and clinical skills simulation environment and provided the context for demonstrating the potential of the integrated AR-BCI prototype solution. The medical education use case scenario illustrated in Figure 1 has served as the primary mechanism for demonstrating and validating the functionality of the prototype. The initial testing and validation of the technology prototype was performed within a small group of approximately 10 health care simulation and multimedia technology experts familiar with a variety of both AR and BCI platforms whose formative inputs helped calibrate the prototype for broader testing and implementation.

After the initial testing by the technology experts, the prototype has been demonstrated to a diverse mix of 2500 potential user representatives, including students, educators, and health care and technology professionals, approximately 600 of whom volunteered to further examine and perform hands-on testing of the integrated AR-BCI solution within the simulation laboratory. The testing volunteers were instructed to don both the HoloLens and NeuroSky Mind Wave 2 devices simultaneously and concentrate their attention on specific 3D objects within the AR environment; for example, “the heart” in order to activate the learning content associated with these objects via neural triggers. All of the volunteers have been able to go through the use case scenarios using the two devices without any problems, which indicates that the technical targets of the integrated AR-BCI system and its individual components have been met under basic operating conditions within the testing environment. The volunteers’ reactions and feedback regarding the overall AR-BCI integration concept and the experience using the prototype have been overwhelmingly positive, ranging from comparisons with science fiction coming to real life to sincere expressions of awe from being introduced to a novel capability that holds tremendous potential for health care applications.

## Discussion

### Principal Findings

This study has successfully proved the concept of integrating commercially available consumer AR and BCI technologies. The results of the described effort present new advancements in the areas of cognitive and computer sciences by providing new capabilities for (1) human-machine interfacing, (2) advanced technology interoperability and Internet of Things networking, (3) multimodal data analytics, and (4) smart and mobile learning technologies in health care. These new capabilities have been realized within the ARNIE technology interface solution that enables interoperability between consumer-grade AR and BCI devices demonstrated within the context of the practical use cases for health professional education and clinical performance support. The proof-of-concept demonstration scenarios involved participation of a broad community of potential end users, including physicians, allied health professionals, medical students, technologists, scientists and researchers, health care administrators who helped validate the integrated AR-BCI technology capability and provided early feedback regarding the prototype, which has been consistently optimistic and supportive in terms of its perceived usability and utility, and also encouraging in terms of its broader testing and implementation in real-life settings.

The resultant new technology solution offers a research test bed and enabler for advancing knowledge and understanding about human-computer interaction in health care by creating an opportunity to connect people (health professionals, patients, and trainees), systems, and data in health care environments through a combination of AR and BCI. This test bed represents a foundation for moving toward plug-and-play integrated AR-BCI technology interoperability, which currently represents a significant barrier to adoption of these new technologies for health care applications.

### Limitations

The study represents an exploratory and developmental technology integration and use case modeling effort, which, thus far, has been successfully demonstrated and implemented in a simulation-based education and research setting, but not in an actual health care delivery environment. The focus of the effort was on technology development and proof-of-concept demonstration of the integrated AR-BCI capability within the realistic use case scenarios. The volunteer participant interaction with the platform was not systematic, and their feedback is considered informal.

### Comparison With Prior Work

The following three major differentiators of the described study from the current state of the science in this area can be distinguished:

#### Expanded Range of Controls for AR Technology Interaction

The world of human-machine interfaces is rapidly transitioning from legacy physical instrumentation to a world driven by gesture, spoken word, and now neural command, which is likely to become far more precise than gesture or spoken word in the future. The results of this study demonstrate an expanded range of AR controls, which includes a neurosensing capability that allows the users to control digital objects in an AR environment using the power of their mind.

#### Consumer-Grade AR-BCI Technologies and Broader Application Focus

A growing body of research suggests the great potential to revolutionize human-computer interaction through the use of emerging AR and BCI technologies, with several early examples starting to demonstrate the applicability of these emerging technologies in a variety of health care contexts, including prosthetic interfaces [18], sensory system rehabilitation [19], behavioral health [20], and others. However, many existing combined AR-BCI implementations use highly specialized technologies and devices that are custom-built for a narrow focus, experimental in nature, and may not be easily adaptable or extensible for wider use, which makes it challenging for them to cross a so-called technology adoption chasm [21] in order to enter mainstream use any time soon. The ARNIE solution brings together the AR-BCI capabilities using the consumer-grade technology and devices available today with the intent to accelerate the transition of this new integrated capability to the consumers in health care in the near future.

#### Technology-Agnostic Solution

The described effort represents a new step toward bringing the AR-BCI technologies together in a device-agnostic way via a novel interface solution that enables communication between consumer-grade wearable AR and BCI devices and provides the user an ability to control digital objects in AR using neural commands. Envisioned to promote effective communication and shared decision-making between health care providers and patients, this new interface represents an extensible and device-agnostic test bed for evaluating future development efforts in this area. Intended to support a wide range of AR and BCI devices, the technology-agnostic integration approach will help promote the adoption of integrated AR-BCI technologies in health care.

### Future Directions

Next steps in this direction would include the development of an expanded set of integrated AR-BCI capabilities and include further testing and implementation of this platform within the health professional education curriculum of the University of Central Florida (UCF) Academic Health Sciences Center. Further research efforts need to advance an understanding about how AR and BCI technologies can be used to (1) facilitate or enhance shared understanding between human agents in health care, (2) support teaching and learning of complex biomedical and medical information, (3) enable strategies to capture mental states and promote metacognition and comprehension in medical trainees and patient populations, and (4) support the identification of neural signatures of complex cognitive, metacognitive, and affective processes during clinical training and performance.

### Broader Impacts

**Figure 4:**
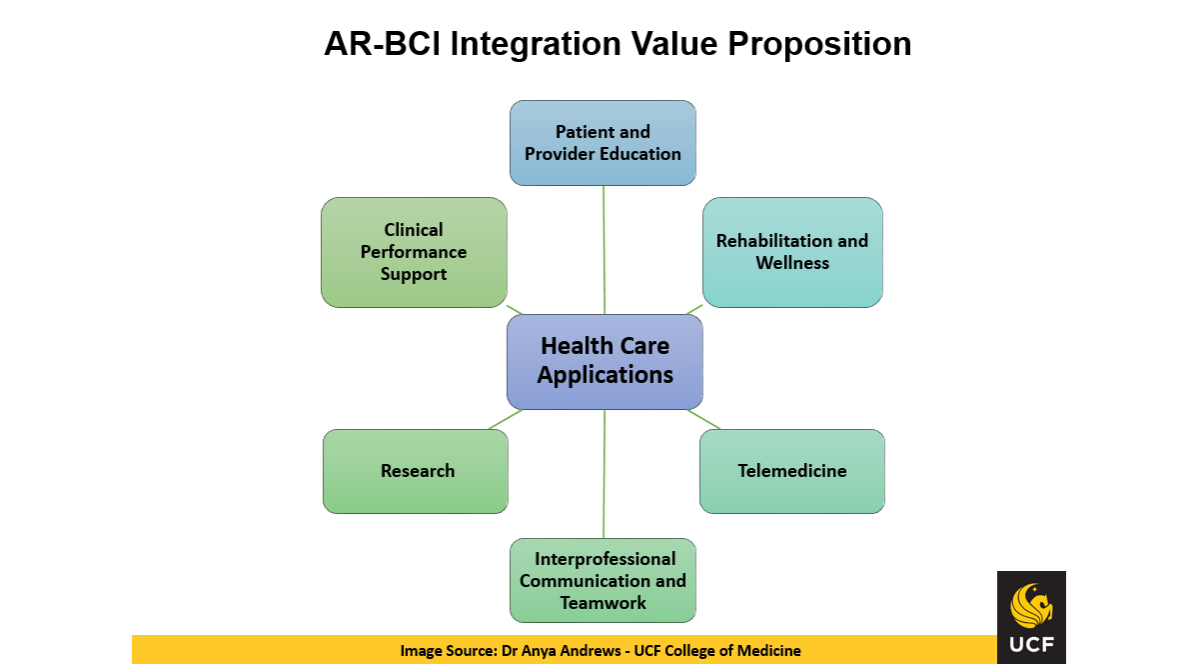
Augmented reality and brain-computer interface (AR-BCI) integration for health care applications: value proposition.

There are many opportunities for broader impacts from the application of integrated AR-BCI solutions in health care, including patient and provider education, physician clinical performance support, interprofessional teamwork, rehabilitation, wellness, telemedicine, and research, as summarized in Figure 4. The results of this study can play an important role in transforming health professions education by enabling the integration of people, systems, and data during the learning process. They can also serve as a powerful enabler for enhanced clinical experiences for patients and providers by improving communication, shared decision-making, and patient health literacy, all of which are associated with patient-centered care [22,23]. The clinical applications of the integrated solution can also augment interprofessional teamwork and help clinical teams maintain situational awareness and shared mental models, which also have a direct impact on patient health outcomes [24]. Additionally, the integration of AR and BCI technologies offers unique opportunities for supporting telemedicine and remote rehabilitation and wellness approaches, which have become particularly important during the COVID-19 pandemic. Finally, the AR-BCI value proposition includes opportunities to advance research in the areas of health care quality improvement and the other health care application areas highlighted in Figure 4.

This described study offers a foundation for future efforts to accelerate the integration of AR and BCI technologies to connect people, data, and systems to enable transformation in health and medicine. As our society continues to become more diverse and global, every effort should be made toward the development of shared understanding in health care [25]. The results of this study and future efforts in this area should help promote shared understanding between health professionals and patients from all walks of life, including underrepresented minorities, patients with limited education background, immigrants, patients in rural communities, and others. A famous quote by a historical figure in medicine, Martin H Fischer, stating that “*In the sick room, ten cents' worth of human understanding equals ten dollars' worth of medical science*,” still holds true today, particularly as patient-centered care remains one of the fundamental aims of the US health care system. Whether it is about communication between providers and patients or between members of an interprofessional team, the development of shared understanding and mental models is an important prerequisite and enabler for shared decision-making.

### Conclusions

Technology-based innovations can serve as a game-changer for supporting patient and provider education, communication, and shared decision-making, which would improve care and engagement of patients and ultimately population health [22]. Through the purposeful integration of multiple disciplines, including cognitive and computer sciences, engineering, and medicine, this study explored the integration of wearable AR and BCI technologies and resulted in a novel integrated AR and BCI technology solution and test bed that brings together two of the most promising technologies to date, both of which have tremendous potential to revolutionize health care. Broad applications for this technology are anticipated in health professions education, clinical performance support, surgery, telemedicine, and patient education. Future research directions in this area should aim to expand the range of current AR-BCI capabilities while addressing existing technical challenges and also generate new evidence on human-computer interaction in health care and health professional education.

## Abbreviations

AR: augmented reality
ARNIE: Augmented Reality and Neurosensing Interaction Environment
BCI: brain-computer interface
EEG: electroencephalogram
HMD: head-mounted display
UCF: University of Central Florida

## References

[1] Vávra P, Roman J, Zonča P, Ihnát P, Němec M, Kumar J, Habib N, El-Gendi A (2017). Recent Development of Augmented Reality in Surgery: A Review. J Healthc Eng.

[2] Lahanas V, Loukas C, Smailis N, Georgiou E (2015). A novel augmented reality simulator for skills assessment in minimal invasive surgery. Surg Endosc.

[3] Alimardani M, Hiraki K (2020). Passive Brain-Computer Interfaces for Enhanced Human-Robot Interaction. Front Robot AI.

[4] Mane R, Chouhan T, Guan C (2020). BCI for stroke rehabilitation: motor and beyond. J Neural Eng.

[5] Mudgal SK, Sharma SK, Chaturvedi J, Sharma A (2020). Brain computer interface advancement in neurosciences: Applications and issues. Interdiscip Neurosurg.

[6] Wen D, Liang B, Zhou Y, Chen H, Jung T (2021). The Current Research of Combining Multi-Modal Brain-Computer Interfaces With Virtual Reality. IEEE J Biomed Health Inform.

[7] Galway L, McCullagh P, Lightbody G, Brennan C, Trainor D (2015). The Potential of the Brain-Computer Interface for Learning: A Technology Review.

[8] Si-Mohammed H, Petit J, Jeunet C, Argelaguet F, Spindler F, Evain A, Roussel N, Casiez G, Lecuyer A (2020). Towards BCI-Based Interfaces for Augmented Reality: Feasibility, Design and Evaluation. IEEE Trans Vis Comput Graph.

[9] Acar D, Miman M, Akirmak OO (2014). Treatment of Anxiety Disorders Patients through EEG and Augmented Reality. Eur Soc Sci Res J.

[10] Mainzer K (2017). From Augmented Reality to the Internet of Things: Paradigm Shifts in Digital Innovation Dynamics. Augmented Reality.

[11] Takano K, Hata N, Kansaku K (2011). Towards intelligent environments: an augmented reality-brain-machine interface operated with a see-through head-mount display. Front Neurosci.

[12] Blum T, Stauder R, Euler E, Navab N (2012). Superman-like X-ray vision: Towards brain-computer interfaces for medical augmented reality.

[13] Barresi G, Olivieri E, Caldwell DG, Mattos LS (2015). Brain-Controlled AR Feedback Design for User's Training in Surgical HRI.

[14] Barsom EZ, Graafland M, Schijven MP (2016). Systematic review on the effectiveness of augmented reality applications in medical training. Surg Endosc.

[15] Davis K, Schoenbaum SC, Audet A (2005). A 2020 vision of patient-centered primary care. J Gen Intern Med.

[16] Graham P, Evitts T, Thomas-MacLean R (2008). Environmental scans: how useful are they for primary care research?. Can Fam Physician.

[17] Sharp A (2009). Requirements modeling with use cases and services. Workflow Modeling: Tools for Process Improvement and Applications Development.

[18] Zeng H, Wang Y, Wu C, Song A, Liu J, Ji P, Xu B, Zhu L, Li H, Wen P (2017). Closed-Loop Hybrid Gaze Brain-Machine Interface Based Robotic Arm Control with Augmented Reality Feedback. Front Neurorobot.

[19] Cervera MA, Soekadar SR, Ushiba J, Millán JDR, Liu M, Birbaumer N, Garipelli G (2018). Brain-computer interfaces for post-stroke motor rehabilitation: a meta-analysis. Ann Clin Transl Neurol.

[20] Luxton DD, June JD, Sano A, Bickmore T (2016). Intelligent Mobile, Wearable, and Ambient Technologies for Behavioral Health Care. Artificial Intelligence in Behavioral and Mental Health Care.

[21] Mitra A, Gupta A (2008). Crossing the Chasm: Business Process to Information Systems. Knowledge Reuse and Agile Processes: Catalysts for Innovation.

[22] National Academies of Sciences, Engineering, and Medicine (2015). Health Literacy and Consumer-Facing Technology.

[23] National Academies of Sciences, Engineering, and Medicine (2018). Improving Health Professional Education and Practice Through Technology: Proceedings of a Workshop.

[24] McComb S, Simpson V (2014). The concept of shared mental models in healthcare collaboration. J Adv Nurs.

[25] National Academies of Sciences, Engineering, and Medicine (2018). Using Technology to Advance Global Health: Proceedings of a Workshop.

